# Early history of the study of bone growth (1722–1875)

**DOI:** 10.1007/s00264-024-06157-w

**Published:** 2024-03-25

**Authors:** Jan Bartoníček, Ondřej Naňka

**Affiliations:** 1 Department of Orthopedics, First Faculty of Medicine, Charles University and the Central Military Hospital, U Vojenské Nemocnice 1200, Prague 6, Czech Republic; 2 Institute of Anatomy, First Faculty of Medicine, Charles University, U Nemocnice 3, 128 00, Prague 2, Czech Republic

**Keywords:** History, Osteology, Long bones, Bone growth, Physis

## Abstract

**Introduction:**

Bone growth is a fascinating process, primarily due to its complexity. Equally engaging is the history of its study, which, however, remains unknown to most anatomists and surgeons.

**Materials and methods:**

A literature search was performed in original publications and historical sources.

**Results:**

The early history of bone growth study may be divided into two periods. Firstly, the experimental one, between 1722 and 1847, which consisted in the study of bone growth by the drilling of benchmark holes into the diaphysis, and examination of growing bones in madder-fed animals. In the course of one century, four French scientists (Henri-Louis Duhamel du Monceau, Marie-Jean-Pierre Flourens, Gaspard Auguste Brullé and Frédéric Léopold Hugueny) and one British researcher (John Hunter) proved experimentally that the longitudinal growth of long bones occurred only at its epiphyseal ends and their final shape resulted from apposition and resorption processes taking place simultaneously both on the periosteal and intramedullary surfaces of the bone. In the second, the microscopic period (1836–1875), the physeal growth cartilage was discovered and described in detail, including its importance for the longitudinal growth of long bones. The first description of growth cartilage was published by a Swiss anatomist Miescher in 1836. Subsequently, this structure was studied by a number of English, German and French anatomists and surgeons. This whole period was concluded by Alfred Kölliker´s extensive study of bone resorption and its significance for typical bone shapes and Karl Langer´s study of the vascular supply of the growing and mature bone.

**Conclusion:**

Research by French, English, German and Swiss scientists between 1727 and 1875 yielded fundamental insights into the growth of long bones, most of which are still valid today.

## Introduction

Bone growth is a fascinating process, primarily due to its complexity. Similarly engaging is the history of its study, spanning almost 300 years, nevertheless unknown to a majority of anatomists and surgeons.

The first, a remarkably detailed historical overview was presented by Kölliker [1] in 1873. In 1874 he was followed by Wegner [2]. Highly informative was the 1917 study by Keith [3]. By contrast, Brash [4], in 1935, mentioned only selected details. A brief outline of the entire history can be found in the books by Lacroix [5] of 1950 and Trueta [6] of 1968. A more detailed overview of this issue was presented by Enlow [7] in 1962. However, no comprehensive up-to-date overview has yet been published in the literature.

### Beginnings of osteology (1627–1733)

One of the first to deal with ossification of the bone was a Flemish anatomist ***Adrianus Spigelius*** ***(1578–1625). ***In his treatise “*De humani corporis fabrica libri X tabulis aere icisis exornati “*, published after his death in 1627 [8], he stated that bone develops from the periosteum and, during growth, the cartilage is gradually replaced by bone. Bone growth was dealt with also by a Dutch anatomist ***Theodor Kerckring (1638–1693),*** in his dissertation thesis “*Osteogenia foetum* “ of 1670 [9]. In 1691, an English anatomist ***Clopton Havers (1657–1702)*** published his study “*Osteologia nova, or some new Observations of the Bones”* [10] where he described, for the first time ever, the microstructure of bone (Haversian osteons) and suggested that the bone grows by intussusception (interstitially). A British anatomist ***Robert Nesbit (1700–1761)*** was the first, in 1733 [11], to point out that the bone may develop both from cartilaginous and membranous anlage. This information, however, fell into oblivion and was “rediscovered” as late as in nineteenth century.

### The first experiments (1722–1847)

The first to approach the issue of bone growth experimentally was the English polyhistor ***Stephen Hales (1677–1761)***. He drilled two holes, one-half an inch apart, into the shaft of the tibia of a growing chicken. Observations made two months later showed that the two holes remained the same distance apart, although the entire bone had increased one inch in length. Based on this finding, Hales concluded that the bone grows in length only at its ends. He published the results of his experiments in 1727 [12] and then abandoned this issue.

Another experimental method came into being by a coincidence. A calico printer in London was feeding his pigs madder-soaked bran (Rubia tinctorum). At that time, madder was used to dye cloth and the refuse served as a feeding mixture. In 1736, he invited for dinner ***John Belchier (1706–1785)***, a young surgeon, and offered him madder-fed pork. The surgeon noticed the ruddy colour of the bones. It was caused by alizarin, contained in madder and staining the newly-formed bone, but not the cartilage. This fact was known as early as in sixteenth century [7], but Belchier was the first to use it experimentally. He conducted several experiments and, still in the same year (1736), he reported on them in the *Philosophical Transactions* [13]. Belchier, however, was not particularly interested in the research of bone growth, as he accepted the opinion of his teacher ***William Cheselden (1688–1752) ***that bones “*grow by the continual addition of the ossifying matter* “.

**Fig. 1:**
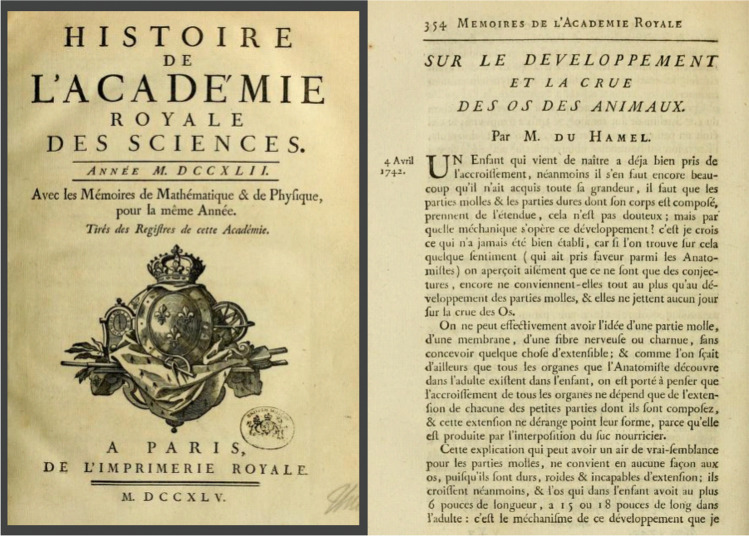
Duhamel´s publication of 1742 [15]

Belchier´s article attracted the attention of a French physician, a naval engineer and botanist ***Henri-Louis Duhamel du Monceau*** ***(1700–1782), ***who made several sophisticated experiments. He fed fowl, turkeys, pigeons and pigs with madder and discovered that only some parts of bones had taken up the red colouring. Subsequently, he used a one-month madder diet combined with a one-month ordinary diet in a young pig. He then killed the animal and found out that the cross-sections of the bone showed alternating white and red circular layers of bone. According to this interpretation, the deep white layer of the bone was formed before the experimental madder diet, the red layer during the madder diet and the superficial white layer during the ordinary diet. Based on this, Duhamel deduced that the transverse bone growth results from periosteal apposition and thus he discovered the osteogenetic function of periosteum, the deepest layer of which he called cambium, by analogy to growing wood. Subsequently, Duhamel made another experiment. He encircled diaphysis of growing bones with a silver wire and found out that after some time, the wire loops had cut into the medullary cavity. He interpreted this finding (wrongly) as a result of expansion of the diaphysis. In another experiment, he drilled holes into the diaphysis of the growing bone, measured their distance, and then filled the holes by silver stylets. After some time, he killed the animal and observed that the distance between the holes did not change. He considered this as an evidence that long bones grow in length only at their ends. Duhamel published his findings serially in 1739–1743 [14–16] (Fig. 1). His major discovery was the osteogenetic function of the periosteum. In spite of this, his experiments were not accepted generally, One of the objections was the fact that he was not a physician.

**Fig. 2:**
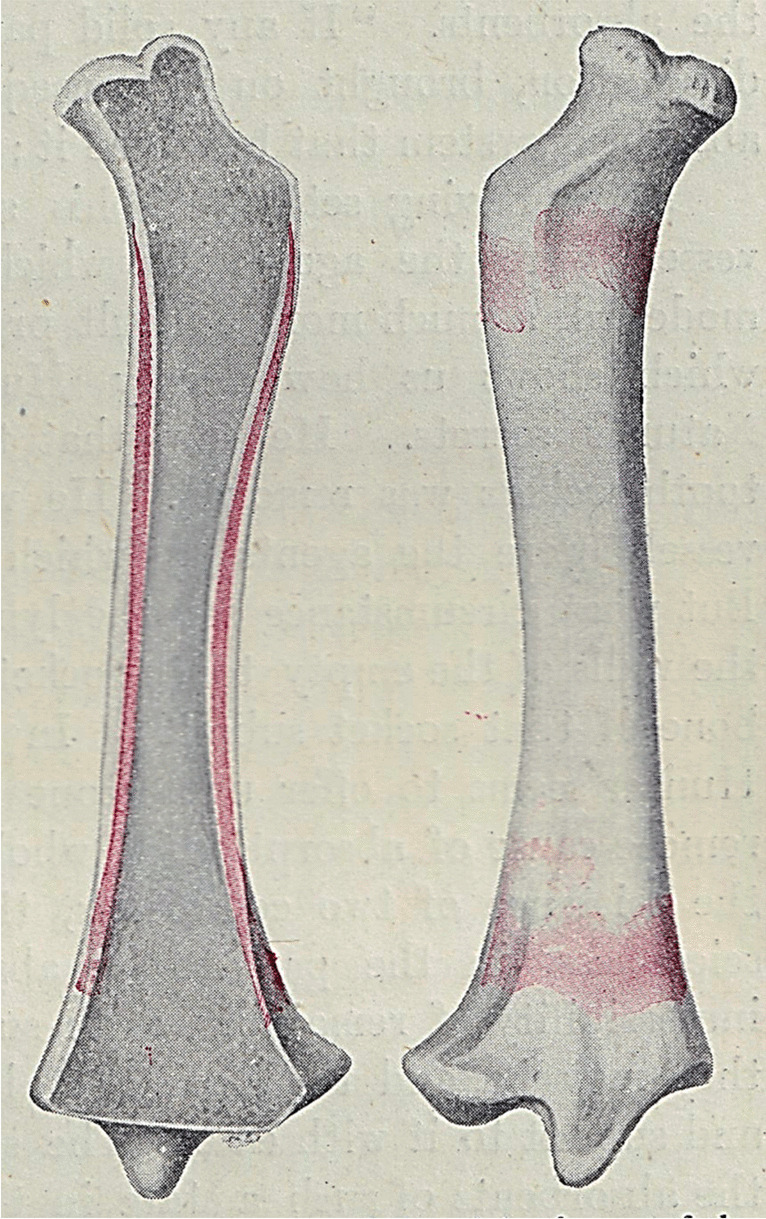
Hunter´s experiment with the growth of the femoral neck. Ruddy color of a bone caused by madder. According to Keith [3]

**Fig. 3:**
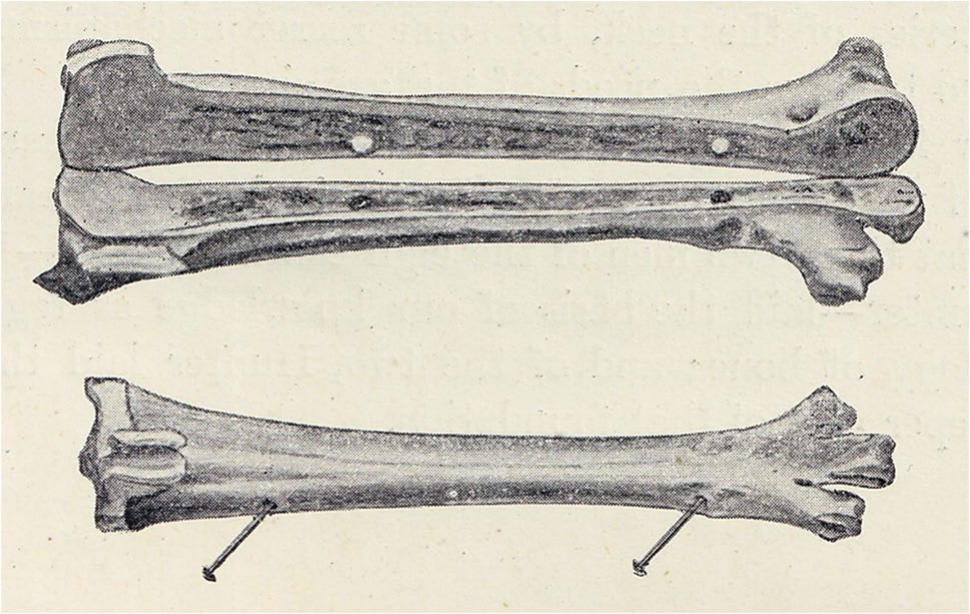
Hunter´s experiment. The distance between the holes drilled into the shaft remained constant during growth, whereas the distance between the shaft holes and the epiphyseal hole increased. According to Keith [3]

Duhamel´s experiments captured the attention of a young physician ***John Hunter (1728–1793)*** from London***,*** a pupil of ***Albrecht von Haller (1708–1777)***. Haller objected to the statement “*that bone is formed only by periosteum* “. In his view, arteries were the depositors and builders of bone and periosteum was merely a vascular covering to serve for the nourishment of bone. Therefore, he devised experiments in order to rebut Duhamel´s concepts [3]. Hunter´s experiments cannot be exactly dated, as his collected works were published posthumously, in 1837 [17, 18]. During the first period, about 1754–1758, he studied mandibular growth with the use of madder-diet. By the way, mandibular growth was intensively debated also in the following centuries [1, 4, 19]. Subsequently, Hunter focused on explaining the growth of the proximal femur and preservation of its shape. He concluded that the femoral neck is being continuously remodeled. In 1764, he confirmed his theory by experiments on young madder-fed pigs (Fig. 2), which showed that the superior surface of the femoral neck during madder period was covered with a new bone, while on the inferior aspect there was no new bone—a clear sign of absorption. Thus, Hunter proved that the final shape of the bone was the result of periosteal apposition and medullary resorption (“*as the bone develops new layers on its outer surface, it loses other layers on its medullary surface “).* Hunter also repeated Duhamel´s experiment with drilling benchmark holes into the bone. He inserted two lead pellets in the tibial shaft of a growing pig. When the tibia was fully grown, their mutual distance remained the same (Fig. 3). However, one sample showed that the drilled holes ran obliquely after completed bone growth, similarly as did the nutrient canals. Hunter did not comment on this fact, and it was explained as late as in nineteenth century (shifting of periosteum along the bone surface during growth in length). Hunter´s opinions on bone growth, primarily his discovery of medullary resorption, were spreading quite slowly in the scientific world. The main reason was the fact that Hunter published them in a fragmented way, and they came into general knowledge only after publication of the whole collection of his works in 1837 [17].

In the following decades, no major discoveries were made. The turning point came in the 1840s. At that time, Duhamel´s and Hunter´s experiments were repeated by a French physiologist from Paris ***Marie-Jean-Pierre Flourens (1794–1867) ***who presented the results for the first time in 1842 in the monograph “*Recherches sur le développement des os et des dents “* [20]. His principal contribution consists in proving intramedullary apposition of the growing bone. Flourens confirmed Duhamel´s and Hunter´s statements that the bone grows in length at its epiphyseal ends, rather than in diaphysis, and that this growth is asymmetric, i.e., that the growth speed of the two ends is not the same.

**Fig. 4:**
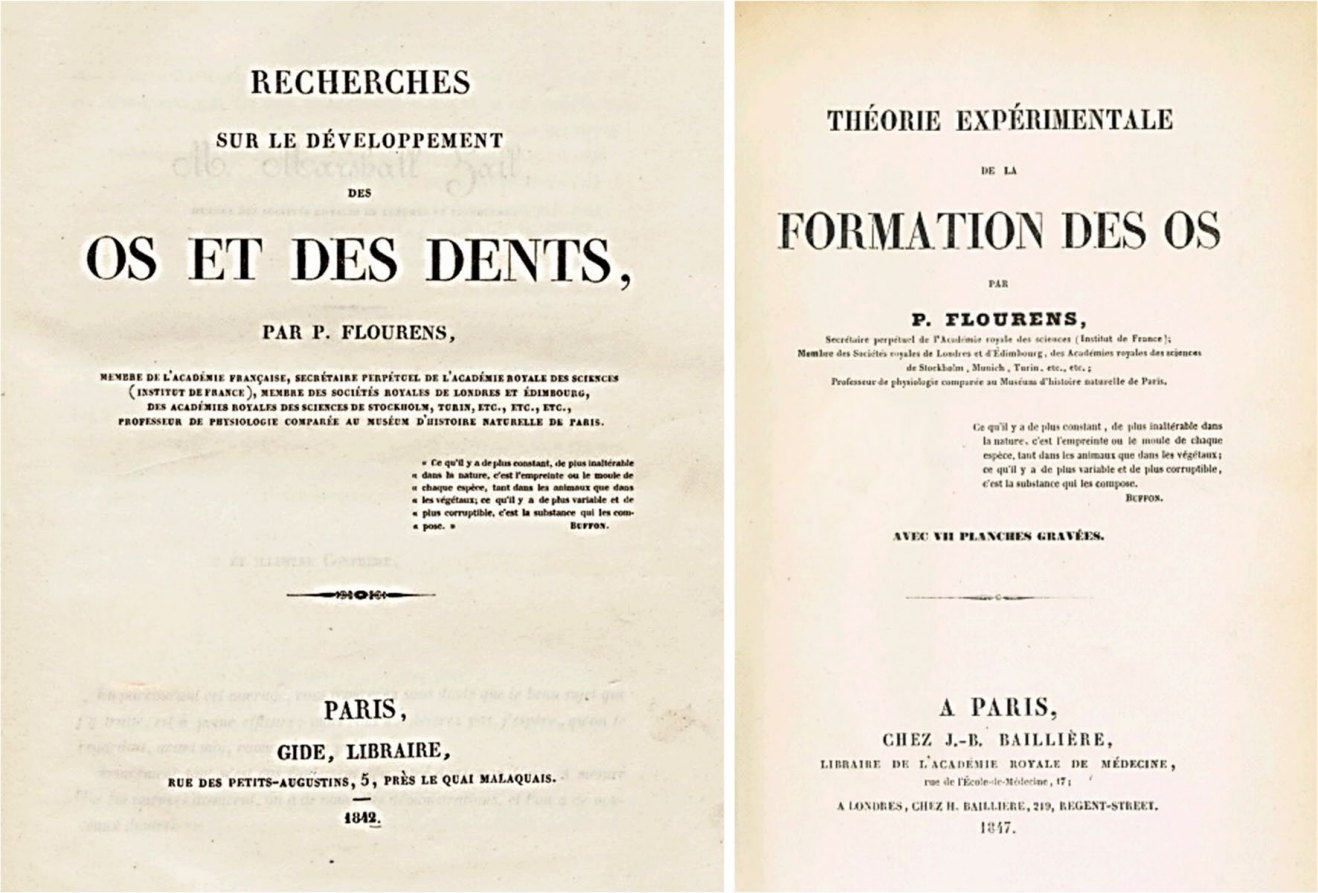
Title page of the Flourens´ publications of 1842 [22] and 1847 [23]

Another significant discovery was made in 1845 by ***Gaspard Auguste Brullé*** ***(1809–1873) ***and ***Frédéric Léopold Hugueny (1815–1896) ***(a natural scientist and a physicist) both working in Dijon in France. They described superficial periosteal resorption at the epiphyseal ends of a newly-formed enchondral bone [21]. This fact was published in a short report in the same year (1845) by Flourens [22], independently of them, who subsequently summarized his results in 1847 [23] (Fig. 4).

Studies of these three French scientists supported rejection of Duhamel´s expansion theory and acceptance of the fact that the bone grows from the epiphyseal cartilage.

Experiments made during 120 years (1721–1847) proved that the long bones grow in length only at their epiphyseal ends and that their final shape results from apposition and resorption processes, which may take place simultaneously both on the periosteal and intramedullary surfaces of the bone. These basic growth and remodeling mechanisms became the subject of much debate in the following decades, and not all prominent authors were able to accept it at that time [24–27]. However, time has clearly proven the correctness of the “Hunter-Flourens theory “ [4–7]. Before that could happen, it was necessary to resolve the question how the bone grows in length and what structure is responsible for it.

### Beginnings of the microscopic study (1836–1875)

**Fig. 5:**
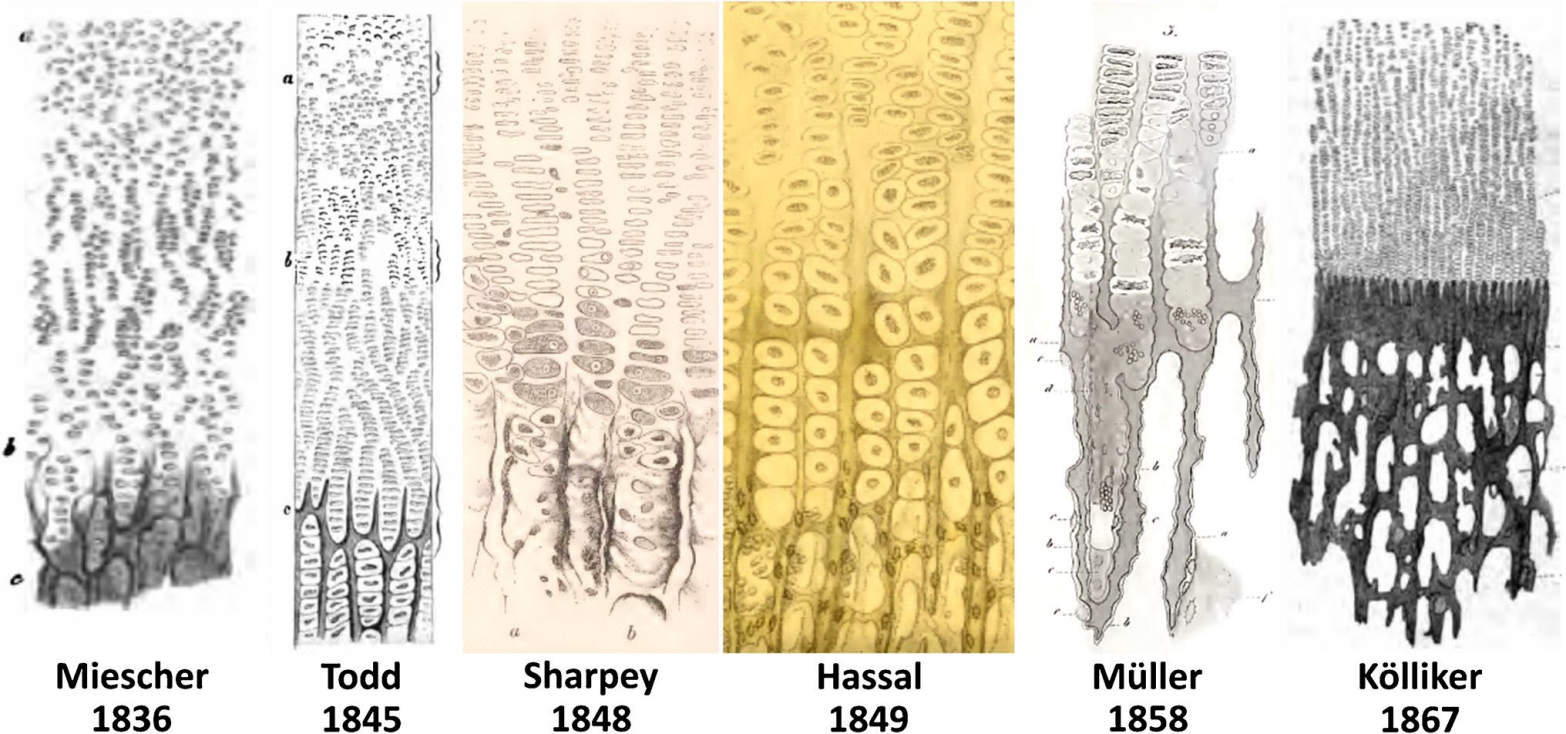
Structure of the growth cartilage according to Miescher [28], Tod and Bowmann [29], Sharpey [30], Hassal [31], Müller [32] and Kölliker [1]

Until the beginning of nineteenth century, the microscopic study of cartilage between the body and epiphyseal end of the long bone had not been addressed. The first microscopic description of the growth cartilage may be found in a Latin dissertation thesis [28] by ***Friedrich Miescher-His (1811–1887)***, a Swiss anatomist from Basel. The dissertation thesis was published in 1836, i.e., prior to development of the cell theory, which is reflected also in the description style. The author states that ossification phenomena can be seen in the epiphysis and diaphysis, but does not specify the ossification structure with a particular term. The ossification zone is formed by two sections (Fig. 5). The section “*ab* “ stands for the cartilaginous epiphysis formed by *corpuscula cartilaginea* (chondroblasts), initially disorganized and later being arranged in columns towards the bone. The section “*bc* “ represents transformation into the bone with dark stripes resembling comb teeth and rows of cartilaginous corpuscles (chondrocytes) between them. As a matter of interest, his son *Friedrich Miescher* *(1844–1895)* is considered to be discoverer of DNA.

Over the next 40 years, a number of fundamental discoveries were made concerning the structure of the growth cartilage and its significance for the longitudinal growth of long bone. Highly valuable in this respect was the newly-formulated cell theory, considering cells as the basic building elements of the human body.

The first researchers to deal with the cellular structure of bone were two Scottish anatomists and surgeons, brothers ***John Goodsir (1814–1867)*** and ***Henry (Harry) Duncan Spens Goodsir (1819–1848) ***in 1845 [33]. The younger of them probably lost his life in the same year during the Franklin´s arctic expedition.

British anatomists ***Robert Bentley Tod (1089–1860)*** and ***William Bauman (1816–1892)*** expanded on Miescher´s study in their textbook of anatomy of 1845 [29]. They dealt with the structure of the growth cartilage in great detail and called it the “*ossifying surface*“(Fig. 5). They describe the growth of bone as follows: *“In the first place, the most important process of growth is continually going on in the cartilage, especially near to*”*ossifying surface”, by the multiplication of the cells; and, in a later stage, by the increase of their dimension… “.*

The Scottish anatomist ***William Sharpey*** ***(1802–1880) ***presented, a very detailed description of “*ossifying cartilage “,* including changes in cartilaginous cells, in the anatomy textbook by *Jones Quain* *(1796–1865)* published in 1848 [30] (Fig. 5). Sharpey’s name is today used as an eponymous term for the fibers fixing periosteum to the bone.

Ossification processes were thoroughly analyzed by a British physician, chemist and microscopist ***Arthur Hill Hassall (1817–1894)***, the author of the first English textbook of histology published in 1849 [31] (Fig. 5).

The founder of modern pathology, and an enthusiastic advocate of the cell theory, ***Rudolf Ludwig Karl Virchow (1821–1902)*** published in 1853 [34] in Würzburg a remarkable study dealing with formation of osteoid, or calcifiable matrix, produced by the osteogenetic cells.

The English surgeons ***John Tomes (1815–1895)*** and ***Campbell de Morgan (1811–1876) ***presented in 1853 a complex overview of the contemporary knowledge of the growth and structure of the bone in an extensive study “*Observations on the structure and development of bone “* [35]. The article was published in an abbreviated form a few months later in the USA [36]. John Tomes is considered to be a pioneer of dental surgery in Great Britain; Campbell de Morgan focused primarily on neoplasia (cancer).

The German anatomist ***Heinrich Müller (1820–1864)*** from Würzburg contributed, in 1858, to the fundamental understanding of the process of calcification of epiphyseal cartilage [32].

The French histologist ***Louis-Antoine Ranvier (1835–1922),*** from Lyon, described, in 1873, circumferential indentation at the periphery of epiphyseal cartilage and termed it *l´encoche d´ossification* (the ossification notch), which is today known as the *notch of Ranvier* [37, 38].

**Fig. 6:**
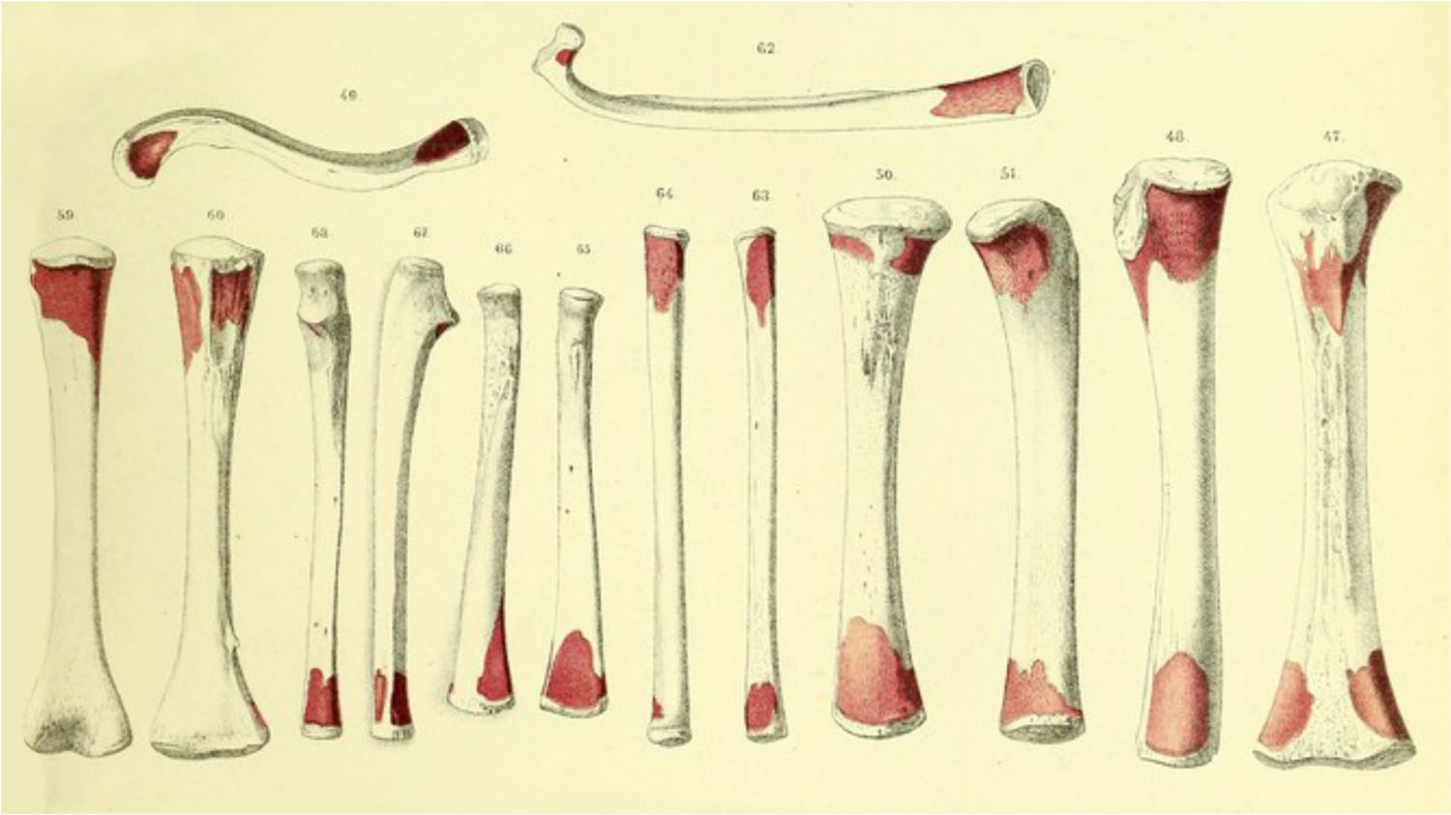
Zones of periosteal resorption (red color) on long bones according to Kölliker [1]

**Fig. 7:**
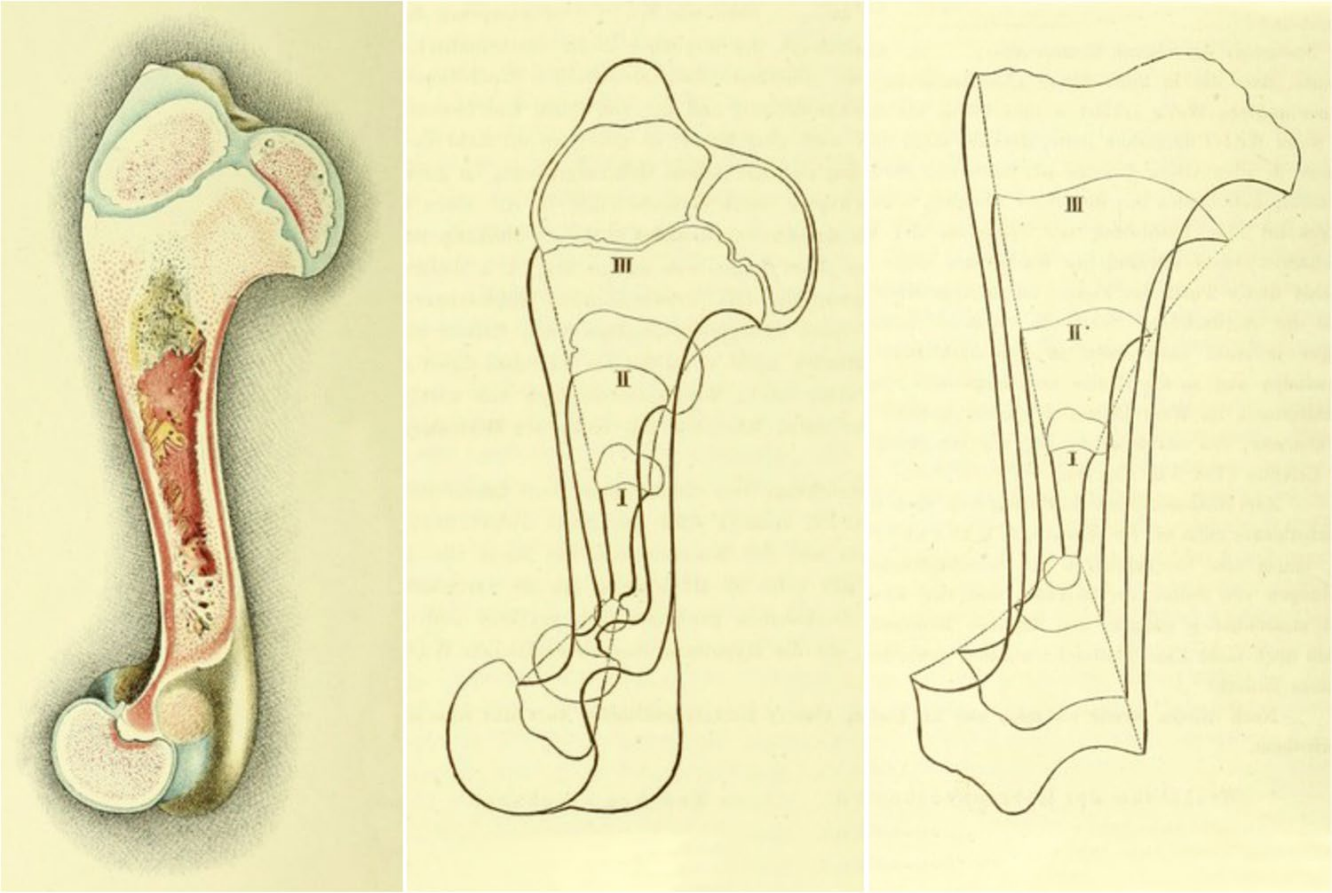
Kölliker´s scheme of remodeling of a growing long bone and drawing of a long bone of a madder-fed pig [1]. The bone modeling scheme respects different activities of the two growth cartilages

In the same year (1873) an outstanding Swiss histologist, ***Albert Kölliker (1817–1905),*** working in Würzburg, published an extensive treatise “*Die normale Resorption des Knochengewebes und ihre Bedeutung für die Entstehung der typischen Knochenformen “* (The normal resorption of bone tissue and its significance for the development of typical bone shapes) [1]. In the introduction, he presented a detailed historical overview of the study of bone ossification and growth. He also described thoroughly the microscopic resorption of bone in the metaphyseal region of long bones (Fig. 6), and thus confirmed the results of experiments of Brullé and Hugueny [21], and of Florens [23]. He called the cells responsible for this process osteoclasts. His research definitively confirmed the significance of resorption during growth of the bone for preservation of its shape (Fig. 7). A radiograph of Kölliker´s right hand, performed by Konrad Wilhelm Roentgen on 23 January 1896, became famous worldwide.

In 1874, a German pathologist ***Friedrich Rudolph Georg Wegner (1843–1917)*** published a comprehensive overview of the growth of long bones [2]. Based on his own experiments, Wegner supported the “Hunter-Flourens´ theory “. In his view, responsible for the longitudinal growth of the long bone was the growth cartilage (das Intermediärknorpel). Wegner [2], together with Kölliker [1] and Virchow [27], strongly objected to the interstitial theory of bone growth advocated mainly by ***Julius Wolff (1836–1902)*** [26], who later became famous for the *Transformation law* (Wolff´s law) [39]. Other supporters of interstitial growth included ***Richard von Volkmann (1830–1889)*** [24] and, partially, ***Carl Hueter (1838–1882)*** [25], the authors of the “*Hueter-Volkmann Law “* [40]. The interstitial theory was supported until the first half of twentieth century when it was definitively disproved [4, 5].

**Fig. 8:**
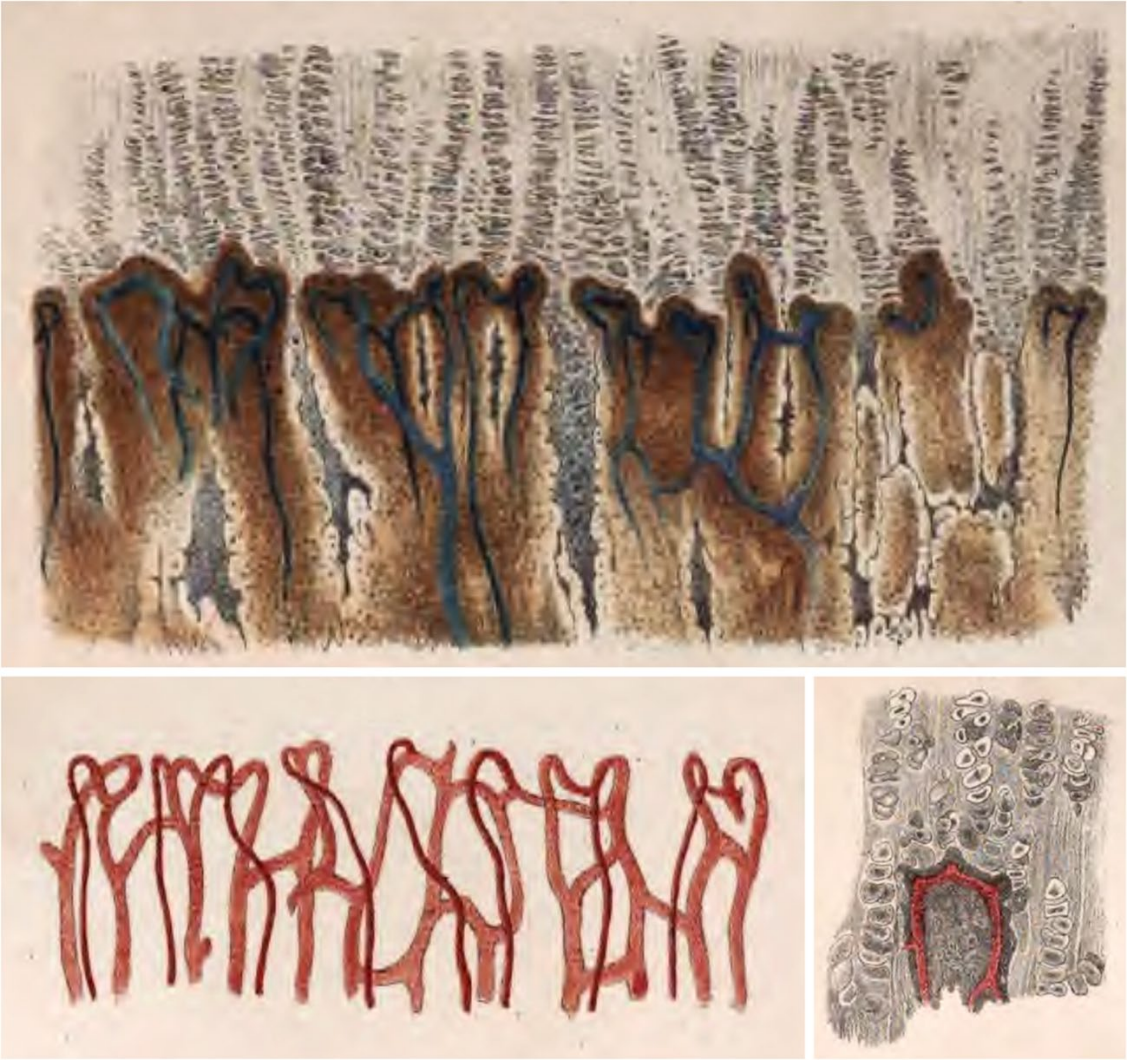
Vessels of the physeal growth cartilage according to Langer [41]

A symbolic end to the "microscopic period" was made by the publication by the Austrian anatomist ***Karl Langer (1819–1887),*** from Vienna, “*Über das Gefässsystems der Rohrenknochen, mit Beitragen zur Kenntniss des Baues und Entwicklung des Knochengewebes “* (On the vascular system of the tubular bones, with contributions to the knowledge of the structure and development of bone tissue) published in 1875 [41]. Langer described there in great detail vascular supply of individual parts of the growing bone, including the growth cartilage (Fig. 8).

## Epilogue

Research between 1727 and 1875 yielded fundamental insights into the growth of long bones, most of which are still valid today. However, these discoveries raised a number of new questions that need to be addressed elsewhere.
